# Mandibular Osteitis Leading to the Diagnosis of SAPHO Syndrome

**DOI:** 10.1155/2018/9142362

**Published:** 2018-06-13

**Authors:** Tomohiro Kikuchi, Hiroyuki Fujii, Akifumi Fujita, Tomoko Sugiyama, Hideharu Sugimoto

**Affiliations:** ^1^Department of Radiology, Jichi Medical University, School of Medicine, 3311-1 Yakushiji, Shimotsuke, Tochigi 329-0498, Japan; ^2^Department of Dentistry, Oral and Maxillofacial Surgery, Jichi Medical University, School of Medicine, 3311-1 Yakushiji, Shimotsuke, Tochigi 329-0498, Japan

## Abstract

Synovitis, acne, pustulosis, hyperostosis, and osteitis (SAPHO) syndrome is a disorder characterized by pustular skin lesions and osteoarticular lesions. Mandibular involvement of SAPHO syndrome is clinically rare, and it is difficult to reach a diagnosis of SAPHO syndrome from only mandibular manifestations. This report describes the case of a 26-year-old woman who presented with mandibular osteitis. Orthopantomogram and computed tomography showed sclerotic change of the right body of the mandible with periosteal reaction without odontogenic infection, which suggested the possibility of SAPHO syndrome. Detailed medical interview found that she had a history of palmoplantar pustulosis treated at a local dermatology clinic and additional bone scintigraphy showed diffuse increased uptake in the right mandible, as well as in the sternum and the sternocostoclavicular joints. She was eventually diagnosed as having SAPHO syndrome. We should consider SAPHO syndrome when we encounter a patient with mandibular osteitis of unknown etiology.

## 1. Introduction

Synovitis, acne, pustulosis, hyperostosis, and osteitis (SAPHO) syndrome is a disorder characterized by pustular skin lesions and osteoarticular lesions proposed in 1987 by Chamot et al. [1]. It is known to present at any age ranging from childhood to adulthood and is characterized by repeated episodes of remission and recurrence. SAPHO syndrome can be difficult to diagnose because of its variable clinical presentation, widespread distribution, and broad spectrum of imaging features [2]. Generally, combinations of osteoarticular and skin manifestations lead to its diagnosis.

Mandibular involvement of SAPHO syndrome is clinically rare and has been reported to comprise 10% of this entity [1]. It is often seen as recurrent mandibular osteitis demonstrating bone sclerosis, mainly involving the body of the mandible [3]. Because a mandibular lesion in SAPHO syndrome is often misdiagnosed as bacterial osteomyelitis, some affected patients received long-term antibiotic therapy combined with surgical procedures and it is rare that the mandibular lesion triggers the diagnosis of SAPHO syndrome [3, 4]. Herein, we report the case of a 26-year-old woman with mandibular osteitis leading to the diagnosis of SAPHO syndrome.

## 2. Case Report

A 26-year-old woman presented to a local dentist due to right mandible pain. She did not complain of any other manifestations, and she had no pertinent past medical history. She was diagnosed with periapical periodontitis, which is an infection of the dental pulp with an apical lesion, of the right lower first molar. She subsequently underwent a root canal therapy. However, she also complained of swelling of the right mandibular region. Since her symptoms had been worsening for 4 months, she was referred to our hospital for further examination and treatment. At our hospital, facial conditions revealed right mandibular swelling and tenderness. Oral conditions showed no percussion and occlusal pain of teeth in the swelling region, and tooth mobility, gum swelling, and gum redness were not seen. Hence, there was no dental infection that could cause osteomyelitis/osteitis. Blood samples were unremarkable with no signs of inflammation.

**Figure 1:**
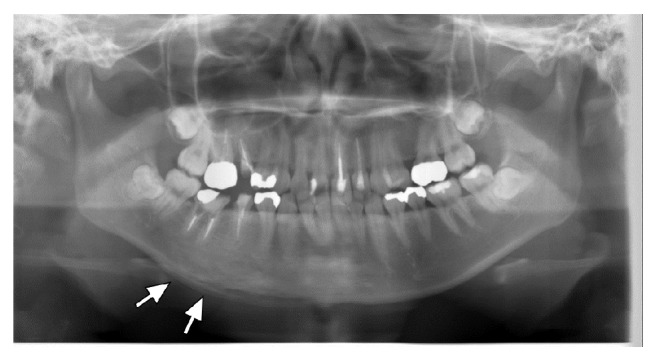
Orthopantomography. Sclerotic changes are seen in the right body of the mandible with periosteal reaction (arrows).

**Figure 2:**
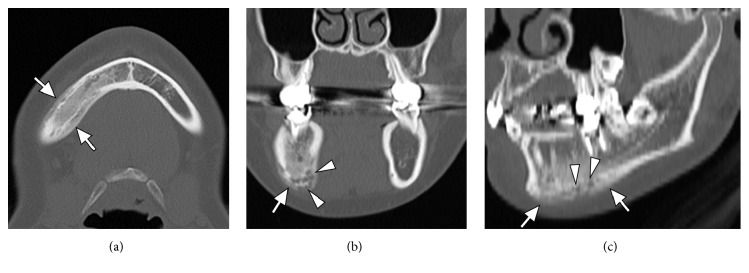
CT scan of the mandible. Axial (a) and coronal (b) and oblique sagittal reconstruction (c) plain CT images show sclerotic change and periosteal reaction in the right body of the mandible (arrows). Spotted osteolysis is also seen mainly in the cortical bone (arrowheads).

**Figure 3:**
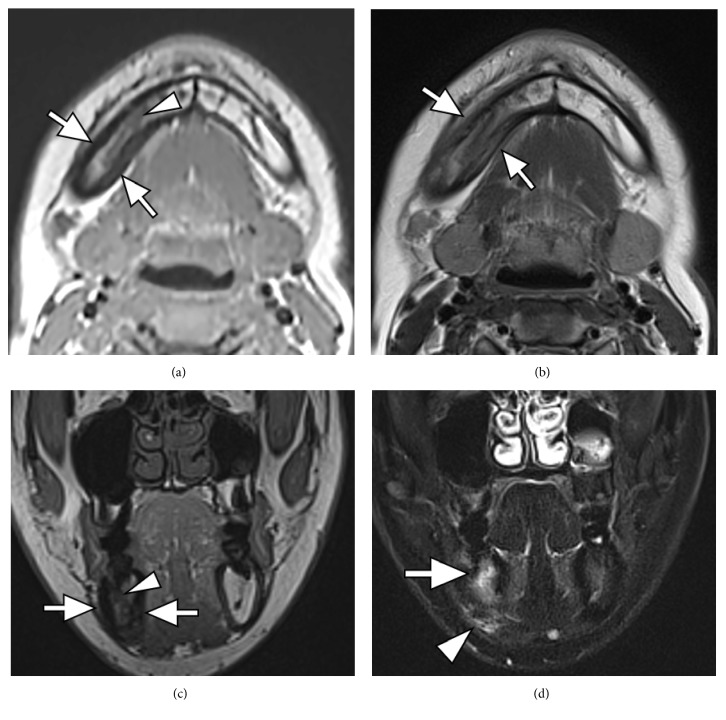
MR imaging of the mandible. Axial (a) and coronal (c) T1-weighted images and axial T2-weighted image (b) show thickening of the right mandible (arrows) and decreased bone marrow fat signal (arrowheads). Coronal short tau inversion recovery image (d) shows high signal intensity in the right body of the mandible (arrow), with perilesional soft tissue swelling (arrowhead).

**Figure 4:**
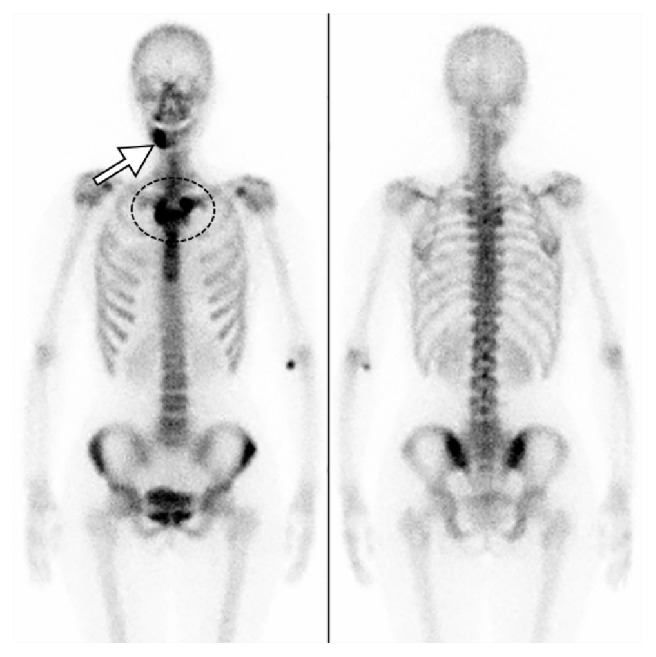
Bone scintigraphy. There is increased tracer uptake in the right mandible (arrow), sternum, and the sternocostoclavicular joints (dotted circle).

Orthopantomogram showed sclerotic change at the right body of the mandible with periosteal reaction (Figure 1). Plain computed tomography (CT) showed sclerotic change at the right body of the mandible with periosteal reaction and spotted osteolysis was seen in the cortex of the mandible (Figure 2). On magnetic resonance imaging (MRI), the right body of the mandible showed low signal intensity on T1-weighted images and high signal intensity on short tau inversion recovery (STIR) images with perilesional soft tissue swelling (Figure 3). Bilateral palatine tonsils and reactive lymphadenopathy were also seen. These findings indicated active mandibular osteomyelitis/osteitis without odontogenic infection. At this time, the possibility of SAPHO syndrome was considered. Detailed medical interview found that she had a history of palmoplantar pustulosis (PPP) for about 1 year, which was treated at a local dermatology clinic. To investigate the presence of other osteoarticular involvement, technetium-99m hydroxymethylene diphosphonate (^99m^Tc-HMDP) scintigraphy was performed, which demonstrated diffuse increased uptake at the right mandible, as well as in the sternum and the sternocostoclavicular joints (Figure 4). These characteristics of clinical and radiological manifestations led to the diagnosis of SAPHO syndrome.

**Figure 5:**
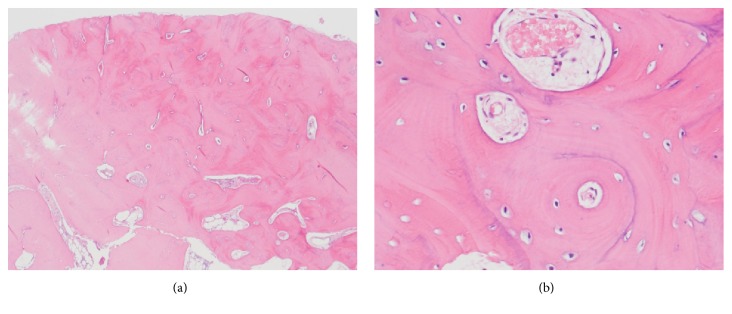
Histopathological findings of the mandible. Enlarged and sclerotic bone trabeculae with little inflammatory cell infiltration [hematoxylin-eosin staining; magnification: 100× (a), 400× (b)].

Tonsillectomy was performed for PPP, and bone biopsy of the right mandible was simultaneously performed. Histopathological examination showed enlarged and sclerotic bone trabeculae with little inflammatory cell infiltration, which was compatible with SAPHO syndrome (Figure 5).

She was treated with salazosulfapyridine 1000 mg/day and nonsteroidal anti-inflammatory drugs as needed, and the pain resolved. Thus far, after a 5-month follow-up period, she has not experienced recurrence of pain.

## 3. Discussion

SAPHO syndrome affects individuals of any age, ranging from childhood to adulthood, and its prevalence has been estimated to be no greater than one in 10,000 in Caucasians and is rarer in Asians [5]. Most patients present with local inflammatory symptoms of pain, swelling, and limitation of movement at the site of active involvement without suppuration [6]. The pathogenesis of SAPHO syndrome remains unknown and several hypotheses, such as infectious, immunological, and genetic mechanisms, have been proposed. An infective cause is a widely accepted theory, and it is described as an autoinflammatory process triggered by low-virulence pathogens [7, 8].* Propionibacterium acnes*, a member of normal flora of the skin and gastrointestinal tract, is occasionally identified from osteoarticular biopsy samples. However, in many instances, biopsy culture is negative and antibiotic therapy is generally ineffective [9]. SAPHO syndrome shares clinical and radiologic features with spondyloarthropathy (SpA), including sacroiliitis, enthesis, paravertebral ossifications, and ankylosis, and many times is associated with psoriasis and inflammatory bowel disease. However, its consideration as a variant of SpA is controversial [10]. Serum rheumatoid factor is usually absent, and human leukocyte antigen-B27 is positive in 4–18% of patients, which is less frequent compared to SpA [7].

The rationale for establishing SAPHO syndrome is that unusual bone involvement is the common denominator among the four skin diseases, such as PPP, acne, psoriasis vulgaris, and generalized pustular psoriasis, by either its radiologic appearance or pathologic features [13]. In 1994, Kahn et al. reported three diagnostic criteria that characterize SAPHO syndrome, which were modified in 2003 (see Table 1) [11, 12]. In many cases, combinations of osteoarticular and skin manifestations lead to the diagnosis of SAPHO syndrome. Our case showed PPP in addition to sterile osteitis of the mandible, and it met the diagnostic criteria.

The most common target site of osteoarticular lesions for SAPHO syndrome in adults is the anterior chest wall, in particular, the clavicles, sternum, and sternoclavicular joints, followed by the sacroiliac region and the spine [14]. Mandibular involvement is relatively rare and has been reported to comprise 10% of this entity [1, 3]. Bone lesions tend to be osteolytic in early stages and osteosclerotic at later stages [14]. Osteosclerotic manifestations include hyperostosis and osteitis, which are chronic inflammatory reactions involving both cortical and medullary bones.

On CT, hyperostosis appears as endosteal and periosteal reactions resulting in generalized cortical thickening and narrowing of the medullary canal. Osteitis appears as increased osteosclerosis involving the trabecular infrastructure of cancellous bone in response to the underlying inflammation [9, 15]. MRI provides better definition of the extent of the inflammatory process in relation to bone marrow edema and soft tissue swelling. Fat-suppressed T2-weighted or STIR images may show high signals on affected sites and are useful to differentiate active lesions from quiescent lesions. Erosions and osteosclerosis are not well depicted, which are well depicted on CT [15]. Bone scintigraphy shows increased tracer uptake in the affected region. In addition, bone scintigraphy is useful because it frequently reveals clinically silent lesions. High tracer uptake in the sternocostoclavicular region yields the so-called “bull's head sign,” with the body of the sternum representing the upper skull and the inflamed sternoclavicular joint with the adjacent clavicles forming the horns [16]. A mandibular lesion also demonstrates the imaging findings described above. The mandibular lesion usually shows unilateral involvement at the body of the mandible [3, 17]. Although it is rare, extension to the temporomandibular joint evolves into ankylosis [18]. In the present case, the right body of the mandible showed sclerotic change with prominent periosteal reaction on CT and showed low signal intensity on T1-weighted images and high signal intensity on STIR images, which were compatible with reported imaging findings.

Some cases with imaging findings similar to mandibular involvement of SAPHO syndrome have been reported under various names, such as chronic recurrent multifocal osteomyelitis, diffuse sclerosing osteomyelitis, and juvenile mandibular chronic osteomyelitis (Garre's osteomyelitis), and some of these cases are considered to be included in SAPHO syndrome [14, 19]. The differential diagnosis for imaging findings of mandibular lesion may include suppurative osteomyelitis, fibrous dysplasia, Paget's disease, osseous neoplasms including osteoid osteoma, osteosarcoma, Ewing's sarcoma, lymphoma, metastases, and Langerhans' cell histiocytosis [14]. It is difficult to differentiate SAPHO syndrome from suppurative osteomyelitis because of its nonspecific findings, such as bone marrow edema and periosteal reaction. Suei et al. reported that SAPHO syndrome revealed osteosclerotic and osteolytic patterns, solid periosteal reaction, external bone resorption, and bone enlargement, whereas suppurative osteomyelitis demonstrated an osteolytic pattern, a lamellated periosteal reaction, and cortical bone perforation [15]. In the present case, the body of the right mandible showed osteosclerotic changes with periosteal reaction and osteolysis mainly in the cortical bone, which was compatible with findings of a previous report [17].

The key to diagnose SAPHO syndrome is to depict both osteoarticular and skin manifestations; however, only the single bone may be symptomatic at disease onset and the skin manifestation does not always occur at the same time [17, 20]. Thus, making a diagnosis of SAPHO syndrome with the mandibular lesion alone as an initial presentation is difficult. In addition, patients may regard skin lesions and mandibular pain/swelling as irrelevant and sometimes skin lesions are hidden. In our case, the medical history of PPP was concealed, and she did not complain of any symptoms except right mandibular pain and swelling when she was first referred to our hospital. We could consider the possibility of SAPHO syndrome from mandibular osteitis because there was no evidence of dental infection, which could cause osteomyelitis.

## 4. Conclusion

We experienced a case of SAPHO syndrome revealed by mandibular osteitis. Although it is difficult to diagnose SAPHO syndrome from only the presence of a mandibular lesion, we should consider the possibility of SAPHO syndrome when we encounter patients with mandibular osteitis of unknown etiology. Detailed medical interview and further imaging examination including bone scintigraphy can lead to the diagnosis of SAPHO syndrome.

## Figures and Tables

**Table 1:** Diagnostic criteria for SAPHO syndrome [11, 12].

Inclusion criteria

(i) Bone and/or joint involvement associated with palmoplantar pustulosis and psoriasis vulgaris, or hidradenitis suppurativa
(ii) Bone and/or joint involvement associated with severe acne
(iii) Isolated sterile hyperostosis/osteitis (adults)^a^
(iv) Chronic recurrent multifocal osteomyelitis (children)
(v) Bone and/or joint involvement associated with chronic bowel diseases

Exclusion criteria

(i) Infectious osteitis
(ii) Tumoral conditions of the bone
(iii) Noninflammatory condensing lesions of the bone

^a^With the exception of *Propionibacterium acnes.*
